# Gender-Based Differences in Health-Related Quality of Life Among Acute Coronary Syndrome Patients: A Cross-Sectional Study

**DOI:** 10.7759/cureus.100554

**Published:** 2026-01-01

**Authors:** Yoshita Gupta, Dinesh K Upadhyay, Abhay Kumar Chaudhary, Grishma Krishnan, Yashika Sapre, Sagnika Bhattacharjee, Ishu Singh, Aashutosh Sinwal, Sonam Pandey, Mudit Bhardwaj

**Affiliations:** 1 Pharmacology, Jaipur National University, Jaipur, IND; 2 Pharmacy Practice, School of Pharmaceutical Sciences, Jaipur National University, Jaipur, IND; 3 General Medicine, Jaipur National University Institute for Medical Sciences and Research Centre, Jaipur, IND; 4 Emergency Medicine, Jaipur National University Institute for Medical Sciences and Research Centre, Jaipur, IND

**Keywords:** acute coronary syndrome, angioplasty, health-related quality of life, mental health, physical health

## Abstract

Background

Acute coronary syndrome (ACS) significantly affects patients’ health-related quality of life (HRQoL). However, limited data exist on HRQoL outcomes following percutaneous transluminal coronary angioplasty (PTCA) in rural Indian populations. This study aimed to assess HRQoL among post-PTCA ACS patients.

Methods

This cross-sectional study evaluated HRQoL in 157 patients with ACS who underwent PTCA at a tertiary care hospital between July 2025 and September 2025. Data were collected using the Short Form Health Survey 12-item (SF-12®v2) questionnaire through convenience sampling. Descriptive statistics, Chi-square tests, and multivariate regression analyses were conducted to explore associations between HRQoL outcomes and sociodemographic variables.

Results

The mean Physical Component Summary (PCS) score was 36.19 ± 8.37, and the Mental Component Summary (MCS) score was 42.19 ± 10.22. Male patients exhibited higher PCS scores (36.54 ± 8.41) and lower MCS scores (40.89 ± 10.20) compared to female patients (PCS: 35.54 ± 8.34; MCS: 43.41 ± 9.51). A significant association was found between sociodemographic variables and HRQoL scores (p < 0.001). Multivariate regression analysis identified age, gender, and occupation as significant predictors of PCS.

Conclusion

ACS substantially impairs HRQoL, particularly among older adults, males, and individuals with lower socioeconomic status. Integrating routine HRQoL assessments and implementing targeted interventions (such as physical rehabilitation, gender-specific psychosocial support, and strategies to mitigate socioeconomic barriers) are essential for improving long-term outcomes and fostering patient-centered care in ACS management.

## Introduction

In India, illnesses affecting the cardiovascular system have become the top contributor to mortality rates since the early 21st century, marking a significant public health concern [1], and socioeconomic factors also have a role in shaping cardiovascular disease outcomes [2]. According to the Global Burden of Disease study, India's age-standardized cardiovascular disease (CVD) mortality rate is 272 per 100,000 people, exceeding the worldwide average of 235 per 100,000 individuals [3]. The increasing prevalence of CVDs is not limited to India but is also evident in several low- and middle-income countries [4,5]. Acute coronary syndrome (ACS) is a crucial subset of coronary heart disease (CHD), encompassing ST-elevation myocardial infarction (STEMI), non-ST-elevation myocardial infarction (NSTEMI), and unstable angina. One-third of deaths in those over 35 are caused by ACS [6].

Health-related quality of life (HRQoL) assesses an individual's ability to function in daily activities as well as their perceived well-being in the physical, mental, and social domains. Functioning denotes the ability to complete specific tasks, whereas well-being refers to subjective emotional and mental states [7,8]. Anchah et al. reported that HRQoL is an independent predictor of mortality and morbidity in ACS patients [9]. In the context of ACS, assessing HRQoL is crucial since ACS treatments aim to improve functional capacity, reduce symptoms, and extend life expectancy, allowing patients to continue participating fully in daily activities [10,11].

Percutaneous transluminal coronary angioplasty (PTCA) has become a key intervention for ACS, with well-documented therapeutic benefits. There is a notable lack of studies evaluating post-procedural health outcomes in Indian ACS patients. This research gap hinders the comprehensive assessment of PTCA's long-term clinical outcomes, particularly its impact on HRQoL. Therefore, changes in HRQoL are influenced by factors such as the prescribed treatment, mental health status, socioeconomic status, the economic burden of lifelong medications, and the patient’s ability to resume normal work life. By addressing this paucity, the current study seeks to provide critical insights into the therapeutic effectiveness and overall impact of PTCA on patient well-being.

## Materials and methods

Aim 

To assess the HRQoL in patients with ACS undergone PTCA.

Study design and setting

**Figure 1:**
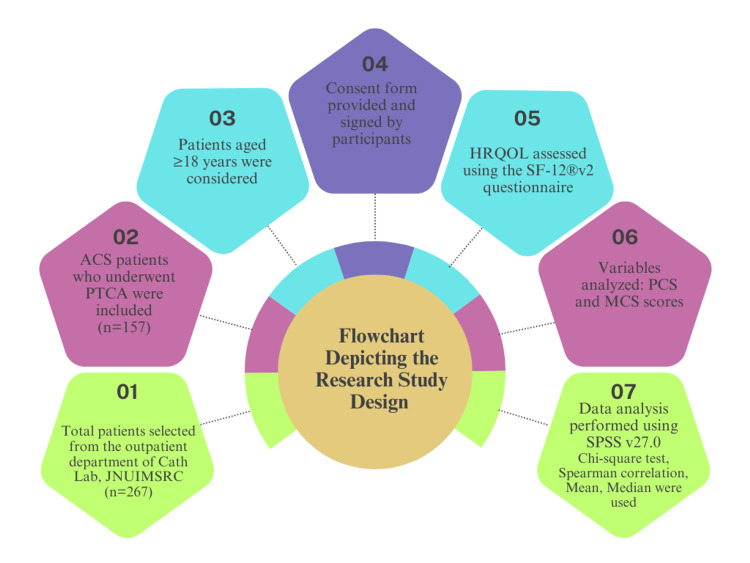
Flowchart depicting research study design. JNUIMSRC: Jaipur National University Institute for Medical Sciences and Research Centre, PTCA: percutaneous transluminal coronary angioplasty, ACS: acute coronary syndrome, HRQoL: health-related quality of life, SF-12: Short Form-12, PCS: Physical Component Summary, MCS: Mental Component Summary, SPSS: Statistical Package for the Social Sciences.

This cross-sectional study was conducted to evaluate the HRQoL among patients with ACS, as shown in Figure 1. The study took place in the Catheterization Laboratory of a tertiary care hospital from July 2025 to September 2025. Ethical approval was obtained from the Institutional Research Ethics Committee, ensuring adherence to ethical research standards.

Ethics approval

The study was approved by the Institutional Ethics Committee of Jaipur National University Institute for Medical Sciences and Research Centre (JNUIMSRC), Jaipur, India (Approval No. JNUIMSRC/IEC/2023/118).

Sample size and sampling technique

The sample size was calculated using Cochran’s formula, with a 95% confidence level and a 5% margin of error, assuming a population proportion of 0.5 to ensure maximum variability. The initial estimated population size was 267, from which a sample size of 157 was calculated. Participants were recruited using a convenience sampling method.

Inclusion and exclusion criteria

Adults aged 18 years or older who provided written informed consent, were diagnosed with ACS, had undergone PTCA, and had been on continuous antiplatelet medication for at least 90 days were eligible for inclusion in the study. Patients with pre-existing heart diseases, cognitive impairments, language barriers, or incomplete data were excluded

Study tools and data collection

**Figure 2:**
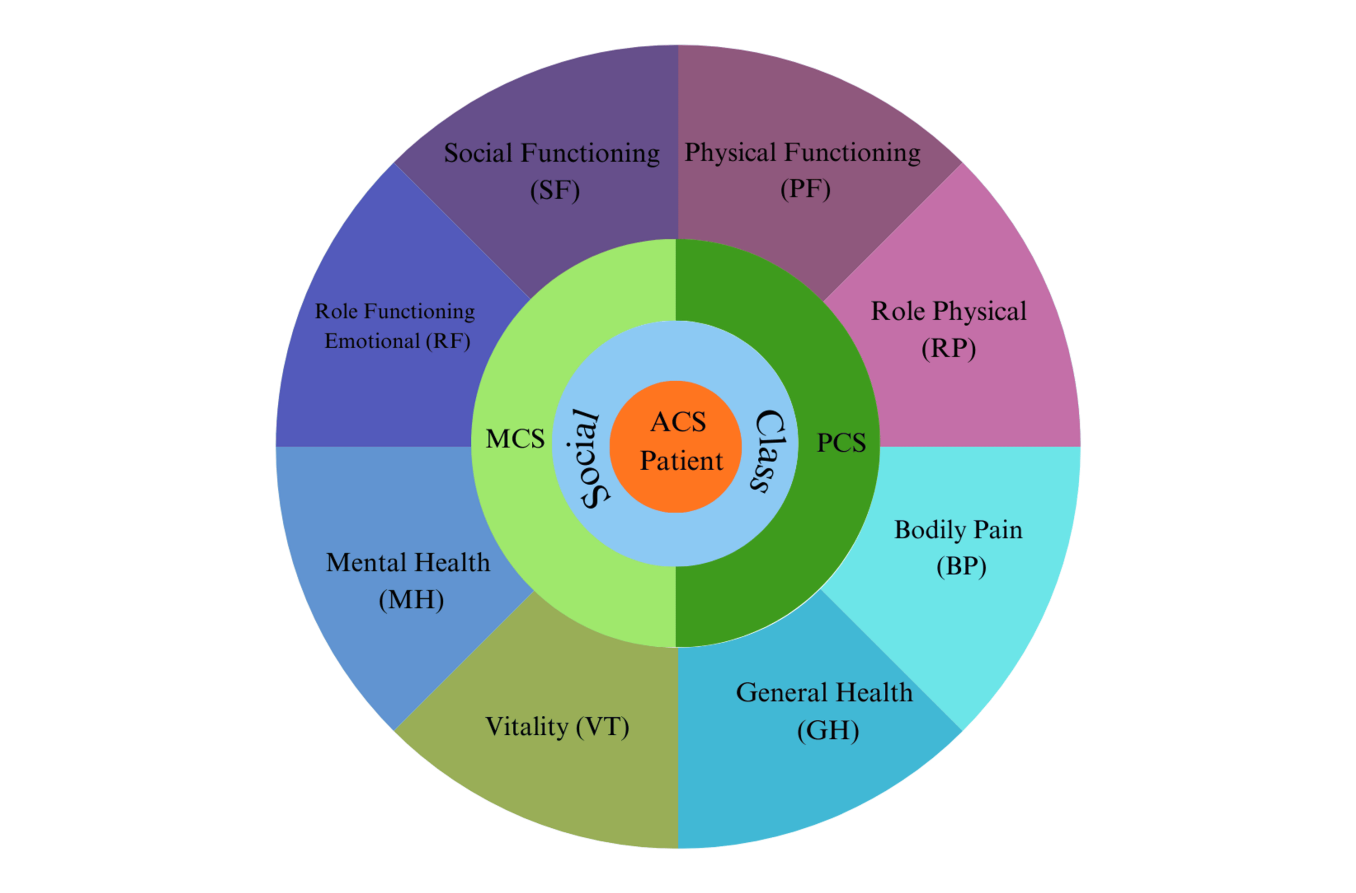
Schematic representation of ACS-induced changes in health-related quality of life. ACS: acute coronary syndrome, PCS: Physical Component Summary, MCS: Mental Component Summary.

In this study, data collection was conducted using three primary tools: a consent form, a demographic questionnaire, and the Short Form Health Survey 12-item (SF-12®v2). The written consent form ensured informed and voluntary participation, and all participant details were kept strictly confidential. The demographic questionnaire was used to collect key participant characteristics. The SF-12®v2, a validated instrument for assessing HRQoL, generates two summary scores: the Physical Component Summary (PCS) and the Mental Component Summary (MCS), which reflect overall physical and mental health, respectively [12]. Additionally, the SF-12®v2 includes eight subdomains (Physical Functioning, Role Physical, Bodily Pain, General Health, Vitality, Social Functioning, Role Emotional, and Mental Health), each assessed through specific Likert scales evaluating frequency, intensity, or degree of limitation (Figure 2). The scoring method for SF-12®v2 uses norm-based scoring for PCS and MCS, with scores standardized to a mean of 50 and a standard deviation of 10. This approach enables comparison of scores, where values above or below 50 indicate above-average or below-average health status. The collected data were entered into IBM SPSS Statistics for Windows, Version 27 (IBM Corp., Armonk, NY) for analysis, with the level of statistical significance set at p < 0.05. Permission to use the SF-12®v2 (SF-12®v2 Health Survey) was granted by IQVIA Inc., and all licensing and permission requests should be directed to IQVIA Inc. at COAsolutions@iqvia.com.

Statistical analysis

Data were entered and analysed using IBM SPSS Statistics for Windows, Version 27 (IBM Corp., Armonk, NY). The Shapiro-Wilk test indicated that the data were not normally distributed; therefore, non-parametric tests were applied.

Descriptive statistics (median and interquartile range for continuous variables; frequencies and percentages for categorical variables) were used to summarize patient characteristics. The SF-12®v2 questionnaire was divided into two components: the PCS and the MCS, which were analysed separately to assess the physical and mental dimensions of HRQoL in post-PTCA ACS patients.

PCS and MCS scores were compared between gender groups using descriptive and inferential analyses. Associations between PCS and MCS scores with various sociodemographic variables (e.g., age, gender, education, occupation) were evaluated using the Chi-square test.

Additionally, multivariate linear regression analysis was performed to identify significant predictors of HRQoL. PCS and MCS scores were treated as dependent variables, while sociodemographic factors served as independent variables. A p-value of <0.05 was considered statistically significant for all tests.

## Results

The study included 157 patients diagnosed with ACS, with a mean age of 57.38 years. Among the participants, 103 (65.6%) were male, and 134 (85.4%) were married. A total of 77 (49.5%) resided in rural areas, and 129 (82.2%) had rural origins. Nearly half of the patients (49%) had no formal education. Regarding occupational status, 59 (37.6%) were unemployed, 38 (24.8%) were workers, and 30 (19.1%) were cultivators.

Table 1 presents the mean scores of the PCS and MCS, along with their standard deviations, stratified by gender. Male patients exhibited slightly higher physical health scores (PCS: 36.54±8.41) but lower mental health scores (MCS: 40.89±10.20) compared to female patients (PCS: 35.54±8.34; MCS: 43.41±9.51). Overall, the mean PCS score was 36.19±8.37, indicating a greater impairment in physical health, while the mean MCS score was 42.19±10.22, suggesting relatively better mental health outcomes in the study population.

**Table 1:** Gender-wise mean scores of physical and mental health of ACS patients. PCS: Physical Component Summary, MCS: Mental Component Summary, ACS: acute coronary syndrome, SD: standard deviation.

Gender	PCS (Mean±SD)	MCS (Mean±SD)
Male	36.54±8.41	40.89±10.20
Female	35.54±8.34	43.41±9.51
Mean±SD	36.19±8.37	42.19±10.22

**Figure 3:**
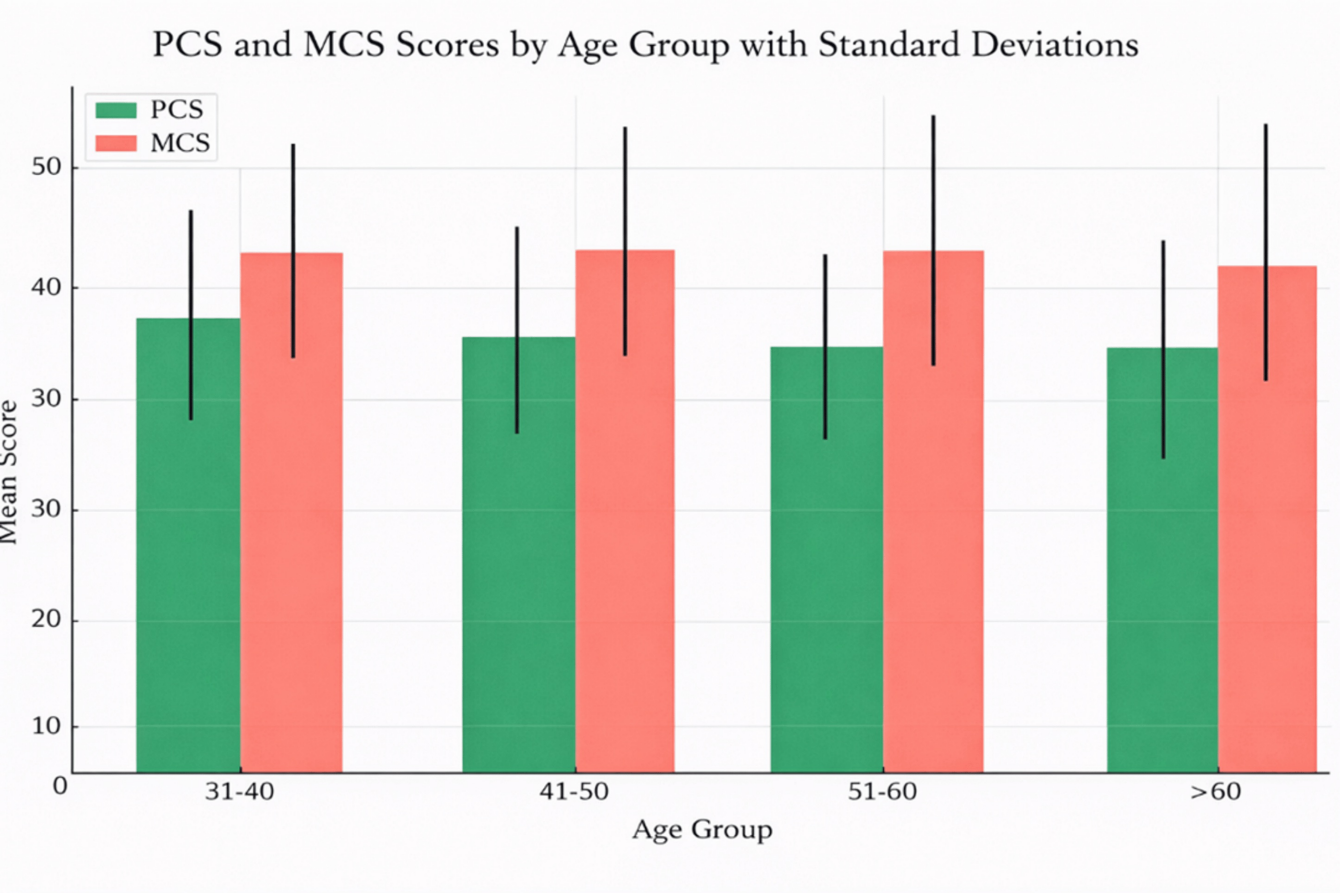
Age-wise comparative analysis of PCS and MCS. PCS: Physical Component Summary, MCS: Mental Component Summary.

Through descriptive analysis, Table 2 summarizes the mean scores and standard deviations of the PCS and MCS from the SF-12®v2 survey, stratified by age groups in the PTCA patient cohort. In the 31-40 year age group, the mean PCS score was 38.92±7.87, and the mean MCS score was 42.85±8.82, as illustrated in Figure 3. Among participants aged 41-50 years, the mean PCS was 36.82 ± SD 8.60, while the mean MCS was 42.68±9.17. For the 51-60-year age group, the mean PCS and MCS scores were 36.01±7.26 and 42.89±8.19, respectively. In participants aged over 60 years, the mean PCS score further decreased to 35.54±9.48, with a corresponding MCS score of 41.18±11.27.

**Table 2:** Patients' age-stratified mean and standard deviation of PCS and MCS scores. PCS: Physical Component Summary, MCS: Mental Component Summary.

Age group (in years)	PCS (Mean±SD)	MCS (Mean±SD)
31-40	38.92±7.87	42.85±8.82
41-50	36.82±8.60	42.68±9.17
51-60	36.01±7.26	42.89±8.19
>60	35.54±9.48	41.18±11.27

Using the Chi-square test, significant differences were observed across various health domains among participants. Physical functioning was notably impacted, with 81 participants reporting limitations in moderate activities (p < 0.001), and 72 reporting difficulty climbing several flights of stairs (p < 0.001), as depicted in Table 3. Role physical outcomes indicated that 128 participants accomplished less than they desired due to physical health limitations (p < 0.001). Regarding bodily pain, 47 participants reported no interference with normal work, while 18 experienced substantial interference (p < 0.001).

**Table 3:** Scale-wise response distribution of SF-12®v2 in ACS patients. SF-12: Short Form-12, ACS: acute coronary syndrome, EVGFP: excellent, very good, good, fair, or poor. *Chi-square test, **Significant at <0.05 (2-tailed test).

Scales	Items	Response choice(s)	n (%)	P-value*
Physical functioning	Moderate activities	Yes, limited a lot	81 (51.59)	<0.001**
		Yes, limited a little	59 (37.57)	
		Not at all	17 (10.28)	
	Climbing several flights of stairs	Yes, limited a lot	72 (45.85)	<0.001**
		Yes, limited a little	64 (40.76)	
		Not at all	21 (13.37)	
Role physical	Accomplished less	Yes	128 (81.52)	<0.001**
		No	29 (18.47)	
	Limited in kind	Yes	125 (79.61)	<0.001**
		No	32 (20.38)	
Bodily pain	Pain-interfere	Not at all	47 (29.94)	<0.001**
		A little bit	40 (25.48)	
		Moderately	37 (23.57)	
		Quite a bit	18 (11.46)	
		Extremely	15 (9.55)	
General health	EVGFP rating	Excellent	12 (7.64)	<0.001**
		Very good	23 (14.65)	
		Good	80 (50.96)	
		Fair	33 (21.02)	
		Poor	9 (5.73)	
Vitality	Energy	All of the time	12 (7.64)	0.006
		Most of the time	23 (14.65)	
		A good bit of the time	39 (24.84)	
		Some of the time	28 (17.83)	
		A little of the time	32 (20.38)	
		None of the time	23 (14.65)	
Social functioning	Social Time	All of the time	31 (19.74)	<0.001**
		Most of the time	19 (12.01)	
		A good bit of the time	16 (10.19)	
		Some of the time	19 (12.01)	
		A little of the time	26 (16.56)	
		Some of the time	46 (29.30)	
Role emotional	Accomplished less	Yes	97 (61.78)	0.003
		No	60 (38.22)	
	Not careful	Yes	94 (59.87)	0.013
		No	63 (40.13)	
Mental health	Peaceful?	All of the time	12 (7.64)	<0.001**
		Most of the time	23 (14.65)	
		A good bit of the time	39 (24.84)	
		Some of the time	28 (17.83)	
		Little of the time	32 (20.38)	
		None of the time	23 (14.65)	
	Blue/sad	All of the time	17 (10.83)	<0.001**
		Most of the time	11 (7.0)	
		A good bit of the time	32 (20.38)	
		Some of the time	36 (22.93)	
		A little of the time	25 (15.92)	
		None of the time	36 (22.93)	

In terms of general health perception, only 12 participants rated their health as excellent, while 80 rated it as good (p < 0.001). Vitality scores revealed that just 12 participants felt energetic all the time, and 39 felt energetic most of the time (p = 0.006). Social functioning was also compromised, with 31 participants reporting that health interfered with their social activities (p < 0.001).

Role emotional limitations were evident, with 97 participants accomplishing less than they would have liked due to emotional problems (p = 0.003) and 94 unable to perform tasks as carefully as usual (p = 0.013). Mental health outcomes showed that only 12 participants felt calm and peaceful all the time, while 32 felt this way only a little of the time (p < 0.001). Likewise, 17 participants felt downhearted and blue all the time, whereas 36 never felt that way (p < 0.001). These findings underscore significant limitations across physical, emotional, and social health domains in the studied population.

Table 4 presents the association between demographic variables and HRQoL in post-PTCA patients, analyzed using the Chi-square test. The analysis revealed significant associations between HRQoL and variables such as age, gender, education, and occupation (p < 0.001), suggesting that these factors notably influence patients' quality of life following the intervention.

**Table 4:** Association of patient’s demographic variables with their HRQoL. HRQoL: health-related quality of life. *Chi-square test, **Significant at <0.05 (2-tailed test). Correlation coefficients indicate the strength and direction of the relationship between each demographic variable and HRQoL.

Demographic variables	P-value*
Age	<0.001**
Gender	<0.001**
Education	<0.001**
Occupation	<0.001**

**Figure 4:**
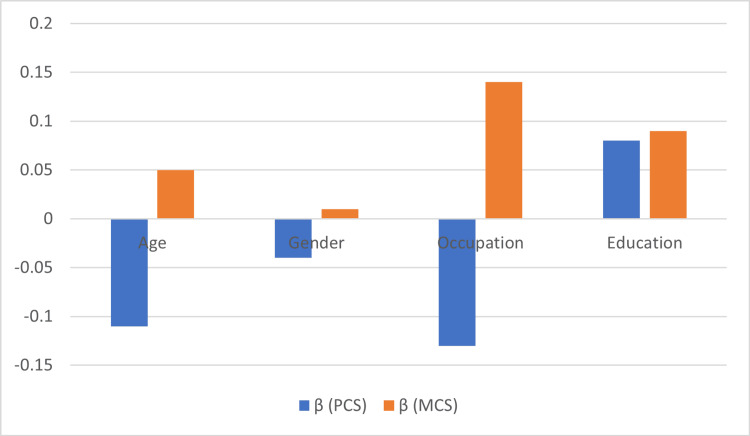
Regression coefficients for predictors of PCS (blue bars) and MCS (orange bars) in ACS patients. PCS: Physical Component Summary, MCS: Mental Component Summary, ACS: acute coronary syndrome.

Table 5 and Figure 4 present the regression coefficients (β values) from a multivariate analysis assessing age, gender, occupation, and education as predictors of PCS and MCS scores. Both age, gender, and occupation displayed negative coefficients in the models, suggesting that being male and employed in certain occupations are associated with lower PCS. The negative impact was more evident among older individuals, reflecting a decline in HRQoL with increasing age. In contrast, educational attainment exhibited a positive influence on both outcomes, with a stronger effect observed on MCS scores, highlighting the link between higher education and improved mental health.

**Table 5:** Contributing predictors in ACS patients HRQoL. PCS: Physical Component Summary, MCS: Mental Component Summary, ACS: acute coronary syndrome, HRQoL: health-related quality of life. *Multivariate regression analysis, significant at < 0.05. Independent variables: age, gender, occupation, and education; dependent variables: PCS and MCS.

Predictors	β (PCS)	t-value	P-value*	β (MCS)	t-value	P-value*
Age	-0.112	-1.342	0.182	0.046	0.548	0.585
Gender	-0.035	-0.387	0.700	0.007	0.074	0.941
Occupation	-0.134	-1.586	0.115	0.141	1.667	0.098
Education	0.08	0.874	0.384	0.093	1.013	0.313

## Discussion

The mean age of the patients in this study was 57.83 years, which aligns with the epidemiological patterns of ACS observed in various international studies [13-15]. A majority of the participants were male (103 (65.6%)) and married, consistent with the higher documented incidence of ACS among men [16]. The mean PCS and MCS scores derived from SF-12®v2 responses were 36.19±8.37 and 44.19±10.22, respectively, indicating that physical health was more adversely impacted than mental health in this patient cohort. These findings are consistent with prior research by Anchah et al. and Seetharam et al., which also noted substantial physical impairments post-ACS, likely due to compromised cardiovascular function and reduced capacity for exertion [9,17].

Female patients demonstrated lower PCS scores compared to their male counterparts, which may be attributed to a greater burden of comorbidities, reduced muscle mass, hormonal influences, longer recovery durations, and heightened psychosocial stress. Factors such as underdiagnosis, differences in pain perception, and socioeconomic disparities may further hinder physical recovery in women [18]. In contrast, McBurney et al. reported no significant gender differences in PCS scores, possibly due to variations in study population characteristics, methodology, or healthcare accessibility [19].

The current study found that MCS scores were lower among male patients, contrasting with findings from Australia and Brazil, where women exhibited poorer mental health outcomes due to higher caregiving burdens and societal pressures [20-21]. In the present context, lower MCS scores in men may be attributed to their increased vulnerability to psychological distress arising from an inability to return to work or fulfil familial and societal responsibilities following the intervention. In Indian society, where men are traditionally the primary breadwinners, such disruptions can lead to substantial socioeconomic and psychological consequences, adversely affecting their quality of life.

Studies from Western countries, however, have frequently reported that females experience a more pronounced decline in HRQoL post-ACS [22-24], a discrepancy potentially rooted in differing gender roles and societal expectations across cultural contexts. These contrasting findings underscore the need for culturally sensitive, gender-specific post-ACS care approaches.

The study further revealed a significant age-related decline in HRQoL, with older patients reporting lower PCS and MCS scores. This trend was especially notable in PCS scores, which showed a stepwise decline across age groups (Table 2). These findings are consistent with existing literature [25,26], attributing reduced HRQoL in older adults to factors such as diminished muscle strength, joint flexibility, and cardiovascular capacity. Among geriatric patients, the decline in both PCS and MCS scores may reflect compounded challenges such as reduced mobility, dependency, and multiple comorbidities.

While MCS scores remained relatively stable among middle-aged patients, they declined significantly among those aged over 60. This decline may result from increased social isolation, lifestyle transitions associated with retirement, and the psychological burden of managing chronic illness. Li et al. also reported similar findings, emphasizing the importance of age-specific interventions to preserve both physical and mental health in elderly ACS patients [27]. Tailored support systems targeting this vulnerable population are crucial to improving post-intervention outcomes.

Subdomain analysis of SF-12®v2 responses revealed significant impairments across physical, emotional, and social dimensions. Limitations in physical functioning (e.g., moderate activities, stair climbing), interference due to bodily pain, and compromised emotional roles (p < 0.001) suggest that ACS significantly restricts functional capacity and emotional resilience. Social functioning was also adversely affected, likely due to limitations in physical activity and engagement. Notably, this study is among the first to present such a detailed subdomain analysis in ACS patients, addressing a gap in existing literature. These findings highlight the necessity of integrated, multidisciplinary approaches that encompass physical rehabilitation, psychological counseling, and social reintegration for holistic recovery.

Significant associations were found between occupational status, education level, and HRQoL scores (p < 0.001), consistent with the findings by Shad et al. [28]. Occupations involving manual labour or high stress were particularly detrimental to health outcomes, underscoring the need for workplace-based interventions and supportive health policies. Regression analysis revealed that education was a significant predictor of both PCS and MCS scores, with higher educational attainment positively influencing outcomes, particularly mental health. These findings support Bahall M., who reported that comorbidities affected PCS more than MCS and that age and depression were the primary determinants of HRQoL [29].

In summary, this study highlights the multifactorial nature of HRQoL in patients with ACS, emphasizing the influence of demographic, socioeconomic, and occupational factors on recovery. Older adults, males, and individuals with lower educational attainment or adverse occupational conditions experienced the most pronounced declines in physical and mental well-being. These insights underscore the need for comprehensive, patient-centred interventions that target high-risk subgroups. Such strategies should include structured physical rehabilitation, integrated mental health support, effective pain management, and socioeconomic assistance. Additionally, the findings highlight the necessity to redefine cardiovascular disease (CVD) risk assessment methods [30] by incorporating HRQoL metrics alongside traditional clinical indicators, ensuring a more holistic understanding of patient outcomes.

However, the study has certain limitations. Being conducted at a single center, the findings may not be fully generalizable to the broader ACS population. The cross-sectional design limits causal inference and reflects HRQoL at only one point in time (after 90 days of post-PTCA and antiplatelet therapy), potentially overlooking longitudinal changes. Additionally, the study's focus on immediate post-PTCA outcomes may not capture the long-term effects of ACS on quality of life. To improve the robustness and generalizability of future ACS research on HRQoL, a multicentric study design with a larger, more diverse sample is recommended. Stratifying patients by access to healthcare programs could reveal disparities in HRQoL, while investigating socioeconomic and demographic factors may identify vulnerable populations needing targeted support. Adding qualitative techniques, such as focus groups or interviews, would enhance quantitative results by offering a greater understanding of patients' actual experiences. By addressing these issues, we can better understand HRQoL in ACS patients and develop patient-centered, more successful therapies.

## Conclusions

This study highlights the substantial impact of acute coronary syndrome (ACS) and percutaneous transluminal coronary angioplasty (PTCA) on the health-related quality of life (HRQoL) of patients in India. The results demonstrate that physical health, as measured by the PCS score, was more adversely affected than mental health. Notably, gender disparities were evident, with male patients reporting poorer mental health outcomes compared to female patients. Age significantly influenced HRQoL, with older patients reporting lower scores, particularly in the physical health domain. Additionally, sociodemographic factors such as occupation and education were associated with variations in HRQoL, suggesting a need for equity-focused care strategies.

Given that patients continue to return for follow-up care post-PTCA, routine HRQoL assessments should be integrated into clinical practice to monitor long-term outcomes. If a patient is found to have compromised HRQoL, targeted counseling and gender-specialized support services should be provided. These findings further highlight the importance of redefining cardiovascular disease risk assessment models to include HRQoL dimensions, thereby enabling more holistic and personalized care. Overall, the study underscores the necessity of patient-centered interventions that not only treat the disease but also address the broader psychosocial and functional challenges patients face after ACS.
